# Concurrent icotinib and definitive radiotherapy followed by detection of an EGFR exon 20 insertion at disease progression in unresectable stage III EGFR S768I-mutant lung adenocarcinoma: a case report

**DOI:** 10.3389/fonc.2026.1902085

**Published:** 2026-08-05

**Authors:** Bingyue Wang, Yangchenxi Wang, Hongyuan Liu, Kaiyue Wang, Xiaona Chang, Sheng Zhang, Zhendong Dai, Chuangyan Wu, Rui Zhou

**Affiliations:** 1 Cancer Center, Union Hospital, Tongji Medical College, Huazhong University of Science and Technology, Wuhan, China; 2 Institute of Radiation Oncology, Union Hospital, Tongji Medical College, Huazhong University of Science and Technology, Wuhan, China; 3 Hubei Key Laboratory of Precision Radiation Oncology, Wuhan, China; 4 Department of Pathology, Union Hospital, Tongji Medical College, Huazhong University of Science and Technology, Wuhan, China; 5 Department of Thoracic Surgery, Union Hospital, Tongji Medical College, Huazhong University of Science and Technology, Wuhan, China

**Keywords:** case report, concurrent radiotherapy, EGFR S768I mutation, icotinib, NSCLC, PACC mutation

## Abstract

Uncommon EGFR mutations account for approximately 10–15% of all EGFR-mutated non-small cell lung cancer (NSCLC), and the optimal management of unresectable stage III disease harboring isolated EGFR S768I mutations remains poorly defined. We report the case of a 55-year-old woman with unresectable stage IIIC lung adenocarcinoma harboring an isolated EGFR S768I mutation. Because platinum-based chemotherapy was discontinued owing to severe gastrointestinal toxicity, she received concurrent icotinib and definitive intensity-modulated radiotherapy (IMRT), achieving a partial response with a progression-free survival of approximately 8 months and only grade 1 oral mucositis. At disease progression, repeat biopsy followed by next-generation sequencing (NGS) identified an EGFR exon 20 insertion (p.V769_D770insSVV) together with a marked reduction in the abundance of the original EGFR S768I mutation. As comprehensive baseline genomic profiling was unavailable, these findings should be interpreted cautiously and may represent either treatment-associated molecular alteration or expansion of a pre-existing low-frequency resistant subclone rather than definitive acquisition of a new mutation. Subsequent treatment with afatinib, ivonescimab plus chemotherapy, and sunvozertinib was selected according to dynamic molecular profiling. This case suggests that concurrent icotinib and definitive radiotherapy may represent a feasible individualized treatment option for carefully selected patients with unresectable stage III EGFR-mutant NSCLC who are unable to tolerate standard concurrent chemoradiotherapy. More importantly, it highlights the value of repeat biopsy and comprehensive genomic profiling for interpreting molecular changes at disease progression and guiding subsequent individualized treatment in patients with uncommon EGFR mutations. However, these observations are derived from a single case and require validation in larger prospective studies.

## 1 Introduction

Definitive concurrent chemoradiotherapy remains the standard treatment for unresectable stage III non-small cell lung cancer (NSCLC), with subsequent consolidation therapy further improving patient outcomes (1). In EGFR mutant-NSCLC, the LAURA study confirmed that osimertinib consolidation significantly prolongs progression-free survival in patients without disease progression after chemoradiotherapy (2). However, this treatment model is primarily based on common EGFR-sensitive mutations, such as exon 19 deletions and L858R, and assumes patients can tolerate platinum-based chemoradiotherapy. The optimal treatment for patients with locally advanced NSCLC who cannot tolerate chemotherapy, particularly those with rare EGFR mutations, remains unclear (3, 4).

Afatinib has demonstrated encouraging efficacy against several uncommon EGFR mutations, including G719X, L861Q, and S768I, and is currently recommended by international guidelines as a preferred first-line treatment for these alterations. However, evidence regarding the efficacy of first-generation EGFR tyrosine kinase inhibitors (TKIs), particularly icotinib, in patients with isolated EGFR S768I mutations remains limited to retrospective analyses and individual case reports (5).Concurrent EGFR-TKI with radiotherapy may balance driver gene inhibition with definitive local radical treatment, providing a potential alternative for patients who are chemotherapy-intolerant (6). Nevertheless, the safety of concurrent TKI and thoracic radiotherapy still requires careful evaluation, particularly with third-generation EGFR-TKIs. Osimertinib combined with thoracic radiotherapy has been associated with a high risk of radiation pneumonitis and interstitial pneumonia (7), suggesting that combining third-generation EGFR-TKIs with radical thoracic radiotherapy may cause substantial pulmonary toxicity.

Increasing evidence indicates that EGFR-mutant NSCLC exhibits substantial intratumoral molecular heterogeneity, with genetically distinct subclonal populations coexisting before treatment initiation. Under the selective pressure of systemic therapy, resistant subclones that are initially present at very low frequencies may gradually expand and become clinically dominant, contributing to disease progression and therapeutic resistance. Recognition of this dynamic clonal evolution has highlighted the importance of comprehensive genomic profiling and repeat molecular assessment throughout the disease course, particularly in patients with uncommon EGFR mutations. Consistent with this biological framework, acquired resistance to EGFR-TKIs reflects ongoing clonal evolution during treatment. Disease progression may result from either the emergence of novel genomic alterations or the selective expansion of pre-existing low-frequency resistant subclones under therapeutic pressure. Consequently, repeat biopsy and comprehensive next-generation sequencing (NGS) at disease progression have become increasingly important for elucidating resistance mechanisms and guiding subsequent therapeutic decision-making (8, 9).

Here, we report a case of unresectable stage III lung adenocarcinoma harboring an EGFR S768I mutation that was treated with concurrent radical radiotherapy and icotinib because chemotherapy was not tolerated. Notably, repeat biopsy at disease progression identified an EGFR exon 20 insertion (p.V769_D770insSVV) together with a marked decrease in the abundance of the original EGFR S768I mutation. Because baseline NGS was unavailable, these findings may represent either treatment-associated molecular alteration or selection of a pre-existing low-abundance subclone under therapeutic pressure. This case highlights the importance of repeat biopsy and dynamic molecular profiling in guiding subsequent treatment decisions. This case suggests that concurrent radiotherapy with icotinib may provide a personalized treatment option for patients with locally advanced NSCLC harboring EGFR S768I mutations who cannot tolerate specific chemotherapy, and highlights the importance of dynamic molecular profiling at progression.

## 2 Case presentation

### 2.1 Initial presentation and diagnosis

**Figure 1:**
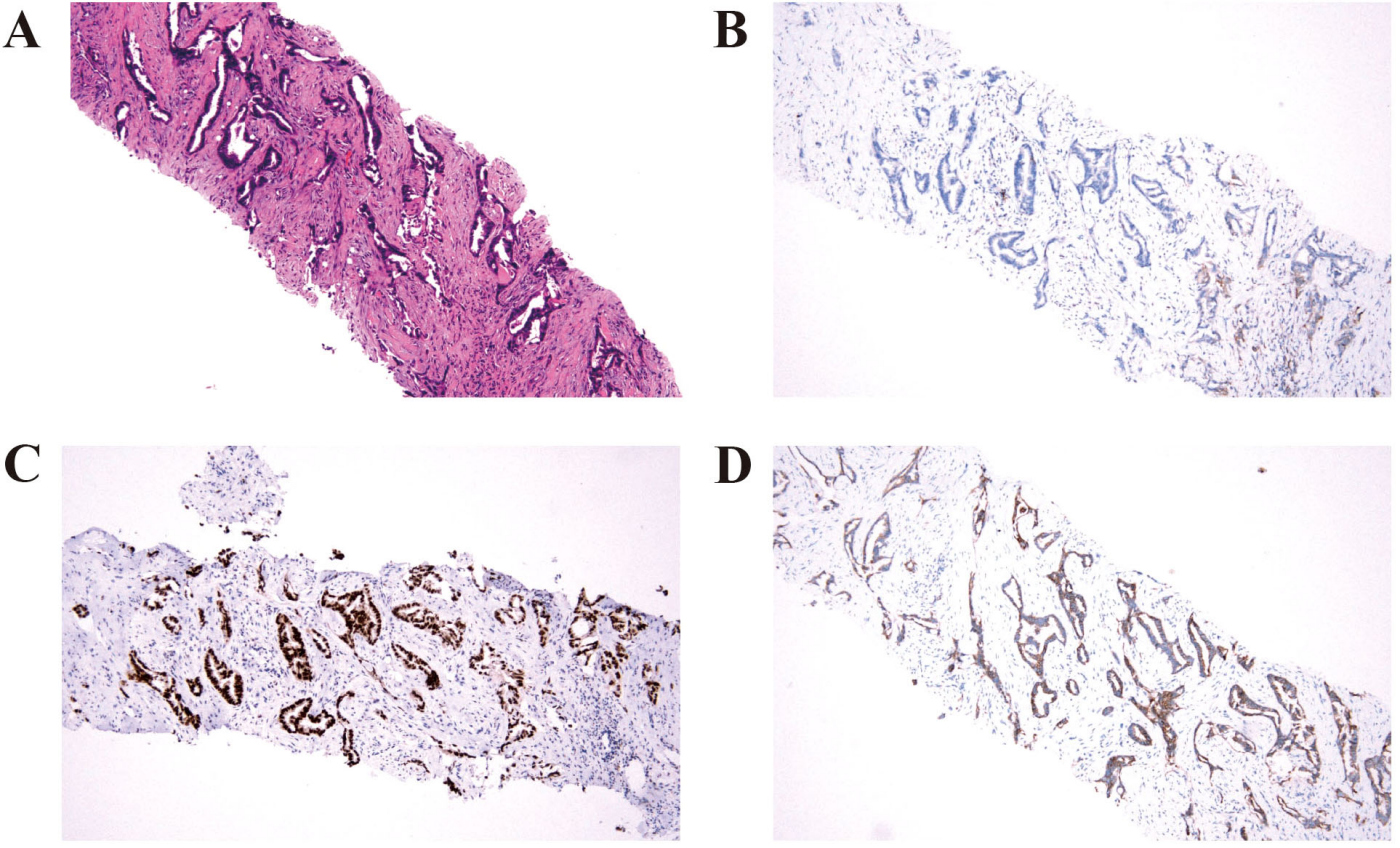
Pathological section shows lung adenocarcinoma. **(A)** HE staining×100. **(B)** PD-L1×100.PD-L1 (MXR006)/Roche Benchmark ULTRA. **(C)** TTF-1×100. **(D)** C-MET×100.C-MET (SP44)/Roche Benchmark ULTRA.

A 55-year-old female presented in July 2024 with a one-month history of cough and right-sided chest pain. Chest CT revealed irregular solid nodules in the middle lobe of the right lung (14 × 9 mm and 23 × 21 mm). Multiple enlarged lymph nodes were identified in the right supraclavicular area and mediastinum, including regions 4R and 7. Initial imaging showed no cervical, axillary, or distant organ metastases. Lung biopsy confirmed lung adenocarcinoma, and the patient was diagnosed with unresectable stage IIIC (cT1cN3M0) NSCLC (**Figure 1**). On August 5, 2024, PCR testing revealed an S768I mutation in EGFR exon 20.ALK, ROS1, RET and KRAS showed wild-type status. Because this PCR assay was designed to detect predefined hotspot mutations rather than perform comprehensive genomic profiling, its ability to identify uncommon EGFR exon 20 insertion variants, including p.V769_D770insSVV, was limited. Consequently, the presence of a pre-existing low-abundance exon 20 insertion subclone at baseline could not be excluded. Unfortunately, insufficient archived tumor tissue precluded retrospective next-generation sequencing (NGS). Tumor tissue from the specimen was subjected to PD-L1 immunohistochemical detection. The PD-L1 test result indicated that the positive score for tumor cells was 5%. PD-L1 antibody clone number: Mianxin PDL1 (MXR006); Detection platform: Roche Benchmark ULTRA. MET immunohistochemistry showed 2+ expression in approximately 20% of cells and 3+ expression in approximately 80% (antibody clone: Ventana SP44).

### 2.2 Chemotherapy intolerance

The patient received two cycles of pemetrexed (500 mg/m²) plus cisplatin chemotherapy (75 mg/m²) on August 13, 2024, and September 7, 2024. Following chemotherapy, the patient experienced treatment-related adverse events, including grade 3 fatigue, grade 2 vomiting, and approximately 5 kg weight loss. Her ECOG performance status declined from 0 to 2. Despite supportive care, symptoms showed limited improvement, and the patient declined further platinum-based chemotherapy. Multidisciplinary evaluation concluded that radical local treatment remained necessary. Although afatinib is generally recommended for EGFR S768I mutations, treatment selection was individualized after multidisciplinary discussion. Considering the patient’s intolerance to cytotoxic chemotherapy, the favorable tolerability and accessibility of icotinib in China, and the limited safety data regarding concurrent osimertinib with radical thoracic radiotherapy. Consequently, concurrent icotinib and radical radiotherapy was initiated.

### 2.3 Concurrent icotinib and definitive radiotherapy

Radical intensity-modulated radiotherapy (IMRT) was delivered using a two-phase sequential approach. During phase I, the gross primary tumor and involved regional lymphatic drainage areas, including the mediastinal and right supraclavicular nodal regions, received 50 Gy in 25 fractions (**Supplementary Figure 1**). Subsequently, a sequential boost was administered to the primary lung lesion, resulting in a cumulative prescribed dose of 60 Gy to the planning target volume (PTV) (**Supplementary Figure 2**). Target delineation followed institutional protocols based on contrast-enhanced planning CT. All dose constraints for organs at risk, including the lungs, esophagus, heart, and spinal cord, complied with institutional planning objectives (**Table 1**). On November 5, 2024, oral icotinib at a dose of 125 mg three times daily was initiated concurrently and was not interrupted during radiotherapy, continuing as maintenance therapy thereafter. Treatment-related adverse events were prospectively assessed according to the Common Terminology Criteria for Adverse Events (CTCAE) version 5.0. Toxicity assessments were performed weekly during radiotherapy and monthly thereafter. Radiation pneumonitis was assessed both clinically and radiologically, with follow-up imaging at 1, 3, and 6 months post-radiotherapy. No grade ≥3 radiation pneumonitis, dermatologic toxicity, diarrhea, hematologic toxicity, hepatic dysfunction, or renal dysfunction was observed throughout follow-up (>6 months). Only grade 1 oral mucositis occurred and resolved with symptomatic treatment.

**Table 1:** Cumulative OAR dose in radiation therapy for organs at risk in the two-phase radical IMRT.

Organ at risk (OAR)	Parameter	Phase I (from Oct 31, 2024)	Phase II (from Dec 20, 2024)
Both lungs	V20 (%)	15.6	6.3
	V5 (%)	36.5	21
	Mean dose (Gy)	9	5
Esophagus	V50 (%)	0	0
	Maximum dose (Gy)	49.9	12.3
Heart	V30 (%)	7.8	1.3
	V40 (%)	3.5	0.5
	Mean dose (Gy)	5.6	5
Spinal cord	Maximum dose (Gy)	29	9.9

### 2.4 Response and follow-up

**Figure 2:**
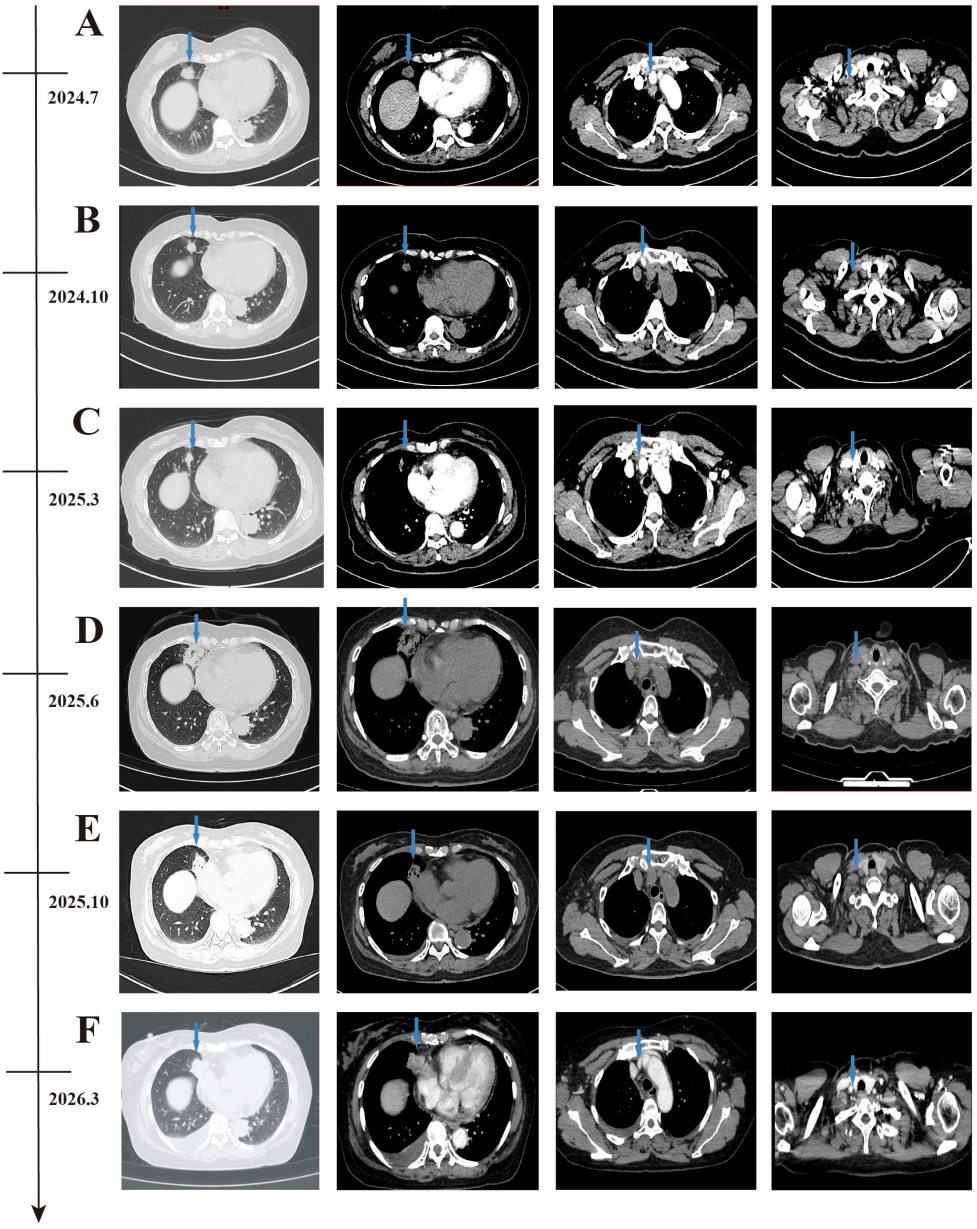
**(A)** Baseline: Computed tomography (CT) scan of thorax, mediastinal and right supraclavicular lymphadenopathy (24*22mm). **(B)** CT scan after chemotherapy (18*16mm). **(C)** CT scan after icotinib plus radiotherapy (16*15mm). **(D)** CT scan of disease progression (July 2025). **(E)** CT scan after afatinib therapy. **(F)** CT scan after pemetrexed + carboplatin + ivonescimab.

**Figure 3:**
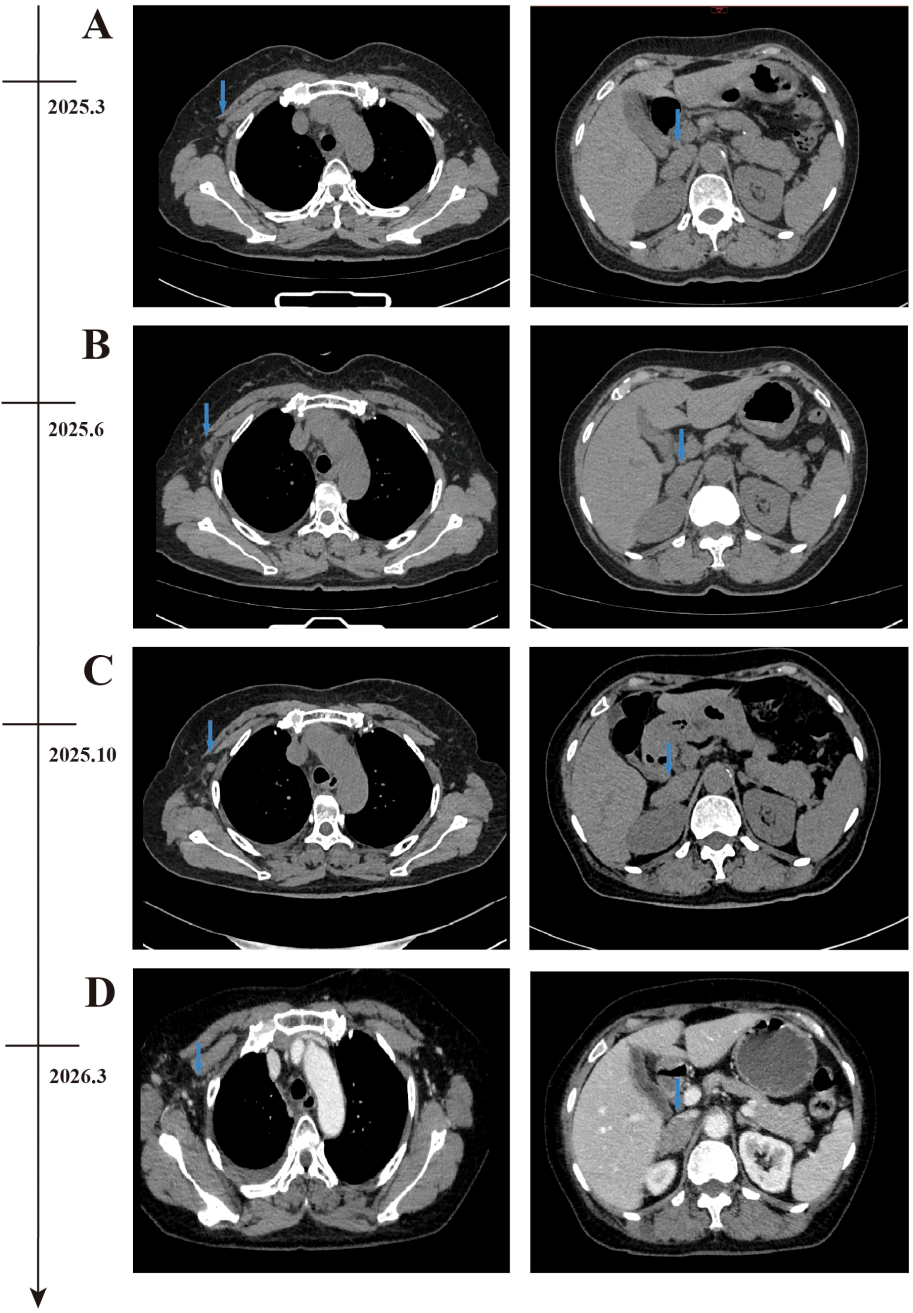
**(A)** Post-treatment CT (icotinib + radiotherapy): right axillary and adrenal metastases. **(B)** Post-treatment CT (icotinib + radiotherapy, 3 months): right axillary and adrenal metastases. **(C)** Post-treatment CT (afatinib, 3 months): right axillary and adrenal metastases. **(D)** Post-treatment CT (pemetrexed + carboplatin + ivonescimab, 3 months): right axillary and adrenal metastases.

Chest CT performed 3 months after treatment showed reduction of the maximum diameter of the right lung primary lesion from 1.9 cm to 1.6 cm (**Figure 2C**). The total diameter of regional lymph node target lesions decreased from 5.2 cm to 2.8 cm, with most other enlarged lymph nodes regressing. Serum CEA level decreased from 3.92 μg/L to 3.11 μg/L. According to RECIST 1.1, serial CT imaging demonstrated a reduction in the sum of target lesion diameters from 71 mm at baseline to 44 mm, corresponding to a 38.0% decrease, thereby fulfilling the criteria for partial response (PR). **Supplementary Table 1** summarizes the target lesion measurements and response classification. The patient continued to benefit from concurrent icotinib and radiotherapy followed by maintenance therapy until disease progression in July 2025, with a progression-free survival of 8.0 months. Follow-up CT in July 2025 showed disease progression with new cervical and axillary lymph nodes and a new right adrenal gland nodule (**Figure 3B**). Notably, the cervical and axillary lymph nodes represented metastatic lesions that were not present at initial diagnosis.

### 2.5 NGS and subsequent treatment

**Figure 4:**
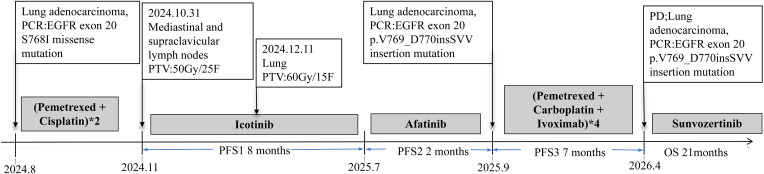
The patient’s treatment process.

Following disease progression, the patient started afatinib at a dose of 40 mg once daily in July 2025. After 1 month of treatment, the primary lesion in the right lung shrunk and remained stable. On September 1, 2025, due to enlargement of the right supraclavicular lymph node, the patient underwent biopsy, and pathology confirmed metastatic adenocarcinoma of lung origin. Targeted NGS demonstrated a marked reduction in the variant allele frequency of the original EGFR S768I mutation together with detection of an EGFR exon 20 insertion (p.V769_D770insSVV). This NGS test detected MET gene copy number amplification (CN: 4.2). Because comprehensive baseline NGS had not been performed, these findings were interpreted cautiously. Specifically, the detected exon 20 insertion may represent either a treatment-associated molecular alteration or therapeutic selection and expansion of a pre-existing low-frequency subclone that was undetectable by baseline PCR testing. From September to December 2025, the patient received four cycles of pemetrexed, carboplatin, and ivoximab, achieving disease stability (**Figure 2E**). Subsequently, the patient experienced a short interruption in anti-tumor therapy. On March 4, 2026, CT showed enlargement of the right lung lesion to approximately 3.2 × 3.4 cm, with multiple metastases in both lungs and the right pleura, and enlargement of the right adrenal gland nodule, indicating disease progression (**Figures 2F**, **3D**). A puncture biopsy of the right cervical mass confirmed metastatic adenocarcinoma of lung origin, and NGS showed persistence of the EGFR exon 20 insertion mutation. Since April 2026, the patient has been receiving sunvozertinib at a dose of 300 mg once daily. As of April 2026, the patient’s overall survival had reached 21 months (**Table 2**; **Figure 4**).

**Table 2:** Chronological timeline of the patient’s clinical course highlighting serial molecular testing and treatment decisions.

Date	Clinical event	Molecular test	Treatment	Response
Jul 2024	Diagnosis			Stage IIIC
Aug 2024	Biopsy	PCR:EGFR S768I		
Aug–Sep 2024	Chemotherapy		Pemetrexed + Cisplatin ×2	Intolerance
Oct–Dec 2024	Definitive IMRT		Concurrent icotinib	PR
Jul 2025	Progression		Afatinib	SD
Sep 2025	Re-biopsy	NGS:S768I↓ + Exon 20 insertion	Carboplatin/Pemetrexed + Ivonescimab	SD
Apr 2026	Progression	Exon 20 insertion persistent	Sunvozertinib	Ongoing

## 3 Discussion

This case illustrates the potential feasibility of concurrent icotinib and definitive radiotherapy as an individualized treatment strategy for selected patients with unresectable stage III EGFR S768I-mutant NSCLC who are unable to tolerate platinum-based chemotherapy. Although concurrent chemoradiotherapy remains the standard of care for locally advanced disease, treatment options for patients with uncommon EGFR mutations who are unsuitable for chemotherapy remain poorly defined. The patient achieved a durable partial response with a progression-free survival of approximately 8 months while experiencing only grade 1 oral mucositis and no grade ≥3 radiation-related toxicities. Although evidence supporting the concurrent use of first-generation EGFR-TKIs with thoracic radiotherapy remains limited, several retrospective studies have suggested that EGFR-TKIs may enhance radiosensitivity by inhibiting DNA damage repair, promoting apoptosis, and suppressing tumor cell repopulation, thereby potentially improving local tumor control without substantially increasing severe toxicity when appropriate radiotherapy planning is employed (10, 11). The selection of icotinib rather than afatinib or osimertinib deserves further discussion. Current international guidelines recommend afatinib as the preferred first-line treatment for major uncommon EGFR mutations, including S768I, based on pooled analyses demonstrating favorable response rates and progression-free survival. However, therapeutic decision-making in clinical practice should also consider patient-specific factors. In this case, severe chemotherapy-induced myelosuppression necessitated an alternative strategy with acceptable tolerability. Given the widespread clinical use, favorable safety profile, and reimbursement availability of icotinib in China, concurrent icotinib plus thoracic radiotherapy was considered a pragmatic individualized treatment approach rather than an alternative to guideline-recommended therapy. Further prospective studies are required to establish its safety and efficacy.

An important finding in this case was the detection of an EGFR exon 20 insertion (p.V769_D770insSVV) at disease progression together with a marked reduction in the abundance of the original EGFR S768I mutation. However, these molecular findings should be interpreted with caution. Because baseline molecular testing was performed using a targeted ARMS-PCR assay rather than comprehensive NGS, it is impossible to determine whether the exon 20 insertion truly developed during treatment or represented a pre-existing low-frequency subclone that underwent selective expansion under therapeutic pressure (12, 13). Increasing evidence indicates that intratumoral heterogeneity and clonal evolution are fundamental biological characteristics of EGFR-mutant NSCLC. Under the selective pressure of EGFR-targeted therapy, resistant subclones that are initially present at very low frequencies may gradually expand and eventually become the dominant tumor population at disease progression (14–16). Although ARMS-PCR remains a sensitive and widely available method for detecting predefined hotspot mutations, it is inherently limited in its ability to identify rare or previously unrecognized genomic alterations, particularly the highly heterogeneous spectrum of EGFR exon 20 insertion variants. In contrast, comprehensive NGS provides broader genomic coverage and has become the preferred approach for identifying uncommon EGFR mutations, resistance-associated alterations, and co-occurring driver events that may influence subsequent treatment selection (17). Therefore, this case should not be interpreted as definitive evidence of treatment-induced molecular evolution but rather as an illustration of dynamic molecular changes observed during disease progression. Repeat biopsy combined with comprehensive genomic profiling at disease progression should be strongly considered whenever clinically feasible. Future studies incorporating serial comprehensive genomic profiling before and after treatment will be necessary to clarify the biological mechanisms underlying such molecular alterations.

Another notable finding was the identification of MET amplification, which is among the most common bypass resistance mechanisms after EGFR-TKI therapy (18). Activation of the MET signaling pathway restores downstream PI3K/AKT and MAPK signaling independently of EGFR, thereby reducing continued dependence on EGFR inhibition (19). Previous studies have reported MET amplification in approximately 5%-15% of patients developing acquired resistance to EGFR-TKIs, although reported frequencies vary according to treatment generation and detection methods. Nevertheless, the coexistence of MET amplification with persistent disease progression strongly suggested activation of an alternative signaling pathway contributing to therapeutic resistance. This observation also highlights the complexity of resistance evolution in uncommon EGFR-mutant NSCLC, where multiple resistance mechanisms may coexist simultaneously.

The sequential treatment strategy adopted in this patient was guided by evolving molecular findings and current evidence-based recommendations. Afatinib was initially selected because international guidelines recommend it as a preferred treatment for major uncommon EGFR mutations, including S768I (5). However, clinical benefit was limited, prompting repeat biopsy and comprehensive genomic profiling. After NGS demonstrated persistent disease together with EGFR exon 20 insertion and MET amplification, subsequent treatment selection was based on contemporary guideline recommendations. Current NCCN, ESMO, and CSCO guidelines recommend platinum-based chemotherapy after EGFR-TKI failure when no validated resistance-directed targeted therapy is available (20, 21). More recently, the phase III HARMONi-A trial demonstrated that ivonescimab combined with platinum-based chemotherapy significantly prolonged progression-free survival compared with chemotherapy alone in patients with EGFR-mutated NSCLC after EGFR-TKI failure, establishing this regimen as an evidence-based treatment option in this clinical setting (22). Therefore, pemetrexed, carboplatin, and ivonescimab represented a biologically rational and guideline-consistent therapeutic choice for this patient and successfully achieved temporary disease stabilization.

Following subsequent progression, repeat NGS consistently demonstrated persistence of the EGFR exon 20 insertion, indicating that this alteration had become the dominant oncogenic driver. Unlike common EGFR-sensitive mutations, exon 20 insertion mutations exhibit intrinsic resistance to first- and second-generation EGFR-TKIs because of structural alterations within the kinase domain (15). Recently developed exon 20-selective inhibitors have substantially improved outcomes for this molecular subgroup. Among these agents, sunvozertinib demonstrated durable antitumor activity and a favorable safety profile in platinum-pretreated patients with EGFR exon 20 insertion-positive NSCLC in the multinational WU-KONG1B pivotal study and has subsequently been incorporated into contemporary Chinese clinical practice guidelines (23). Accordingly, initiation of sunvozertinib represented a molecularly matched precision treatment strategy based directly on serial genomic profiling.

Several limitations should be acknowledged. First, this is a single-patient observation, and therefore the efficacy and safety of concurrent icotinib with definitive radiotherapy cannot be generalized. Second, comprehensive baseline NGS was unavailable, preventing definitive determination of whether the exon 20 insertion represented true treatment-induced molecular evolution or expansion of a pre-existing resistant clone. Third, MET amplification was detected only by NGS without orthogonal confirmation using FISH, limiting assessment of its clinical significance. Finally, follow-up after initiation of sunvozertinib remains relatively short, and longer observation is required to evaluate the durability of response.

In summary, this case highlights three important clinical messages. First, concurrent icotinib and definitive radiotherapy may represent a feasible individualized treatment option for carefully selected patients with unresectable stage III uncommon EGFR-mutant NSCLC who cannot tolerate standard chemoradiotherapy. Second, serial biopsy with comprehensive NGS provides critical information regarding molecular evolution and resistance mechanisms that cannot be captured by baseline hotspot testing alone. Finally, dynamic adaptation of systemic therapy according to evolving genomic alterations—including the transition from uncommon EGFR mutation-directed therapy to chemotherapy plus ivonescimab and ultimately to exon 20-targeted therapy—illustrates the central role of precision oncology in the management of uncommon EGFR-mutant NSCLC.

## 4 Patient perspective

The patient initially suffered severe fatigue and weight loss from chemotherapy. She tolerated concurrent icotinib and radiotherapy well, experiencing only mild mouth sores. She valued the repeated biopsies and genetic testing, which she felt provided clear guidance for each treatment decision, helping her understand the changing nature of her disease and maintain confidence in the personalized care she received.

## 5 Conclusion

This case describes the clinical course of a patient with unresectable stage III lung adenocarcinoma harboring an isolated EGFR S768I mutation who achieved durable disease control with concurrent icotinib and definitive radiotherapy after being unable to tolerate platinum-based chemotherapy. At disease progression, repeat biopsy and comprehensive next-generation sequencing identified an EGFR exon 20 insertion (p.V769_D770insSVV) together with a marked reduction in the abundance of the original EGFR S768I mutation. Because comprehensive baseline genomic profiling was unavailable, these molecular findings should be interpreted cautiously and may reflect either treatment-associated molecular alteration or expansion of a pre-existing low-frequency resistant subclone rather than definitive acquisition of a new mutation. This case highlights the importance of repeat biopsy and comprehensive genomic profiling in interpreting molecular changes at disease progression and supporting individualized treatment selection in patients with uncommon EGFR mutations. Although concurrent icotinib and definitive radiotherapy appeared to be feasible in this patient, the clinical value of this strategy and the molecular evolution of uncommon EGFR-mutant NSCLC require further investigation in larger prospective studies.

## Data Availability

The original contributions presented in the study are included in the article/**Supplementary Material**. Further inquiries can be directed to the corresponding authors.

## Supplementary material

Supplementary Figure 1 Target: mediastinal and bilateral supraclavicular lymph node drainage areas. Prescription: PTV 50Gy/25F. PTV, The planning target volume; GTV, gross tumor volume; CTV, clinical target volume.

Supplementary Figure 2 Target: primary right lung lesion (sequential boost).Prescription: PTV 60Gy/15 F. PTV, The planning target volume; GTV, gross tumor volume; CTV, clinical target volume.

Supplementary Table 1 RECIST 1.1 target lesion assessment. Overall RECIST 1.1 response: Partial response (PR) (≥30% decrease in sum of target lesion diameters).
