# Supplementary material for: Concurrent icotinib and definitive radiotherapy followed by detection of an EGFR exon 20 insertion at disease progression in unresectable stage III EGFR S768I-mutant lung adenocarcinoma: a case report
Source: Front Oncol. 2026 Aug 5;16:1902085. doi: 10.3389/fonc.2026.1902085 (PMC13485718; doi:10.3389/fonc.2026.1902085)
Supplement: Supplementary file 1 [file Table1.docx]

**Supplementary Table S1.RECIST 1.1 target lesion assessment.Overall RECIST 1.1 response: Partial response (PR) (≥30% decrease in sum of target lesion diameters)**

| Target Lesion | Baseline (sum of longest diameters, mm) | Follow-up at 3 months (sum of longest diameters, mm) | Change (%) |
| --- | --- | --- | --- |
| Right lung primary tumor | 19 | 16 | −15.8% |
| Regional lymph node target lesions (4R, 7, right supraclavicular) | 52 | 28 | −46.2% |
| Total | 71 | 44 | −38.0% |
| RECIST | PR | | |
